# Nursing and the Sustainable Development Goals

**DOI:** 10.1590/1518-8345.0000.4038

**Published:** 2023-11-03

**Authors:** Andreia Jorge Silva da Costa, Gisele Câmara, Paulo Jorge Nogueira, Maria Adriana Pereira Henriques

**Affiliations:** 1 Instituto de Saúde Ambiental, Faculdade de Medicina, Universidade de Lisboa, Lisboa, Portugal.; 2 Escola Superior de Enfermagem de Lisboa, Centro de Investigação, Inovação e Desenvolvimento em Enfermagem de Lisboa, Lisboa, Portugal.; 3 Universidade de Lisboa, Faculdade de Medicina, Laboratório Associado TERRA, Lisboa, Portugal.

**Figure:**
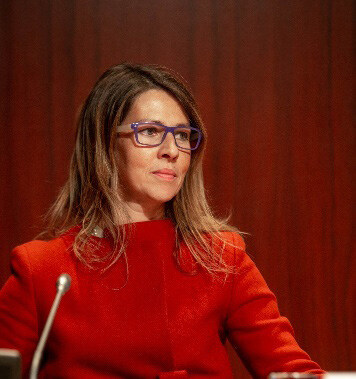


**Figure:**
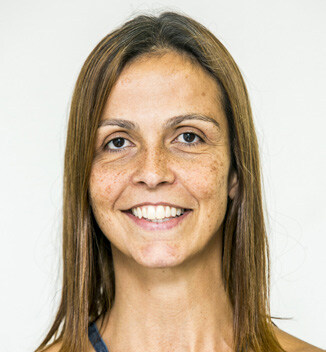


**Figure:**
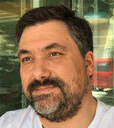


**Figure:**
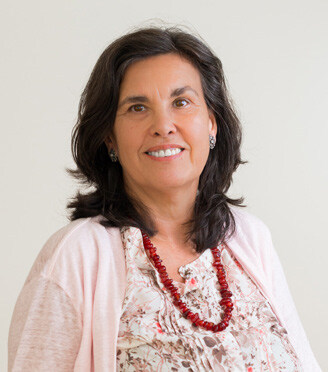


In recent years, several worldwide events have also exerted an impact on the Nursing profession. Climate change and the COVID-19 pandemic are but some examples of complex issues that created a scenario marked by disruption and uncertainty at the global level in which Nursing is also involved. The United Nations Sustainable Development Goals (SDGs) offer a roadmap to address these global concerns, but require broader reflection as a way forward ^(^ 1 ^)^. 

In a scoping review carried out in 2020, based on the 35 articles analyzed the authors reached the conclusion that, in their individual practices, nurses seem to feel disconnected from the SDGs and unable to relate the goals to their clinical role, which implies the need to promote awareness and education about the goals. For the review authors, in a broader view of the profession, Nursing can also contribute to research and policies in relation to the SDGs, strengthening its position in terms of being an active voice and developing a prominent role in achieving the goals. Nurses and the Nursing profession at large have opportunities to contribute more meaningfully to the SDGs, starting with increased awareness through education and a commitment to research and participation in local and global decision-making. It is important to note that only four of the 35 articles analyzed concerned original papers and that two only dealt with Goalv 3: “Health and Well-being” ^(^ 2 ^)^. 

That same year, the article entitled “Nursing and Sustainable Development Goals in the COVID-19 global reality: The state of science and a call to Nursing for leadership” was published. In it, the authors listed some facts that showed the importance of Nursing in achieving the SDGs, namely: nurses’ enormous representativeness in the health sector since, together with midwives, they add up to almost half of the global health workforce; the relevance and recognition of nurses’ role in the COVID-19 pandemic; and the 2017 statement by the President of the International Council of Nurses on the urgency of nurses’ participation at the highest policy- and decision-making levels ^(^ 3 ^)^. 

The review carried out by the authors identified some needs in the discipline and in the Nursing profession referring to the SDGs, namely:

- Training - Nursing curricula and training revised to include content on thev SDGs;
- Research - development of research studies on innovative and disruptive Nursing, documenting advances towards achieving the SDG 2030 Agenda;
- Practice - Nursing practice in line with the SDGs;
- Policies - developing responsive and proactive Nursing policies that envision what is required to achieve the SDGs ^(^ 3 ^)^.

Recognizing that education about the SDGs is a fundamental basis for future nurses to actively contribute to achieving the goals, we support the proposal to incorporate the SDGs into Nursing curricula based on Freire’s pedagogy ^(^ 4 ^)^, arguing that a critical approach to education is necessary to create the necessary transformation so that Nursing students are duly trained on the SDGs, empowering future nurses for action, whether at the research, practice or policy level ^(^ 1 ^)^. 

Although not exclusively, training in Community Nursing represents an opportunity for an initiative of this nature. By approaching the social determinants of health, it is possible to bridge the gap between Nursing and the SDGs, expanding future nurses’ views and also of those currently involved in initiatives of this nature, regarding their role in achieving the SDGs.
